# An Unusual Case of Acute Scrotal Pain

**DOI:** 10.7759/cureus.45221

**Published:** 2023-09-14

**Authors:** Thomas Damrow, Ryan Bellinger, Judy Lin, Jennifer A Walker

**Affiliations:** 1 Department of Emergency Medicine, Baylor Scott & White All Saints Medical Center, Fort Worth, USA; 2 Department of Emergency Medicine, Burnett School of Medicine at Texas Christian University, Fort Worth, USA

**Keywords:** idiopathic spinal subdural hematoma, covid-19, focal neurologic deficit, spontaneous spinal hematoma, emergency department, groin pain, testicular pain, scrotal pain

## Abstract

Patients frequently present to the emergency department with complaints of scrotal or testicular pain. Generally, there is an algorithmic approach to workup, which includes assessment for torsion, infection, or vascular causes, and musculoskeletal causes of pain are also sometimes considered. Spinal cord pathology, however, is less often explored as a cause of testicular pain. Here, we present a case of a 45-year-old man with end-stage renal disease and hypertension who presented with acute testicular pain. After a comprehensive workup, however, the source of pain was not initially found. Progression of the patient’s symptoms led to the diagnosis of spontaneous spinal subdural hematoma. This atypical presentation of a rare diagnosis is also interesting due to the patient’s concomitant diagnosis of an otherwise asymptomatic COVID-19 infection. While our case represents an atypical combination of clinical features, it also illustrates the importance of continued vigilance and ongoing workup when patients present with severe pain and unclear causes of their symptoms.

## Introduction

Scrotal pain is a relatively common complaint and comprises 0.5% of visits to the emergency department (ED) [1]. Etiologies are numerous and can include surgical emergencies such as trauma, deep tissue infection, aortic dissection, and testicular torsion, but can also include less serious causes such as musculoskeletal pain [2,3]. Importantly, delays in these more emergent diagnoses can lead to high morbidity and mortality. Evaluation of scrotal pain includes physical exam and often includes testicular ultrasound and urinalysis [1-3] but can expand into workups for other causes, such as aortic dissection, radicular pain, groin strain, or mild trauma in the appropriate context [2]. 

Spinal cord pathology causing isolated scrotal or testicular pain is less commonly considered. A few case reports have described scrotal pain associated with lumbar disc herniation [4,5]. When physical examination, imaging, and urinalysis do not reveal testicular or scrotal pathology, neurogenic causes should be considered. The anterior scrotum is innervated by L1 and L2 branches including the genital branch of the genitofemoral nerve and the superior branch of the ilioinguinal nerve. The posterior scrotum is innervated by S2 and S3 branches including the pudendal nerve and the posterior femoral cutaneous nerve [4,6]. Central or peripheral injury or compression of these spinal cord levels or nerve roots can therefore produce scrotal pain. Processes related to compression or inflammation of these nerve roots can include abdominal aortic aneurysms, retroperitoneal masses, conus medullaris tumors, and appendicitis [4].

In this report, we describe a case of spontaneous spinal subdural hematoma (sSDH) initially presenting as a cause of isolated, acute testicular pain. sSDH is a rare phenomenon [7-10] that most often is associated with coagulopathy [7,8,11]. Only a few reports have documented spinal cord bleeding with coronavirus disease 2019 (COVID-19) infection [12-15], and we found only one other report of sSDH associated with COVID-19 [16]. We describe a unique case of testicular pain caused by sSDH which is one of the first cases of sSDH associated with an otherwise asymptomatic COVID-19 infection. While the presentation and etiology of our case are rare, it is an important reminder to consider spinal cord pathology in the differential diagnosis of testicular pain.

## Case presentation

A 45-year-old man with hypertension and end-stage renal disease (ESRD) on hemodialysis three times per week presented to the ED with complaints of abrupt-onset and worsening, right-sided testicular pain that was not relieved by over-the-counter medications. Because of his ESRD, he was anuric. He denied missing his most recent dialysis session which was in the previous 24 hours. His blood pressure was usually difficult to control, and he reported taking labetalol, amlodipine, hydralazine, and clonidine for his hypertension. He did not endorse missing any doses of medications. He denied any history of trauma, fever, back pain, abdominal pain, numbness, or weakness.

His vital signs on arrival were as follows: BP 177/114 mmHg; HR 68 bpm; RR 18; SpO_2_ 94% on room air; temperature 36.8°C (98.2 F). On physical exam, he appeared uncomfortable and anxious. His lungs were clear to auscultation bilaterally, and cardiovascular exam revealed regular rate and rhythm without murmurs, rubs, or gallops. Pulses were documented as palpable in all extremities. Abdominal exam was soft with mild right, lower quadrant tenderness to palpation without rebound or guarding. No abdominal masses or hernias were noted. His genitourinary exam revealed a normal penis with right scrotal tenderness to palpation with normal lay and cremasteric reflex without swelling or overlying erythema. His left testicle was normal. Neurologic exam was documented as normal, including musculoskeletal strength. He was able to ambulate into the ED. No other pertinent findings were documented on his exam.

**Figure 1:**
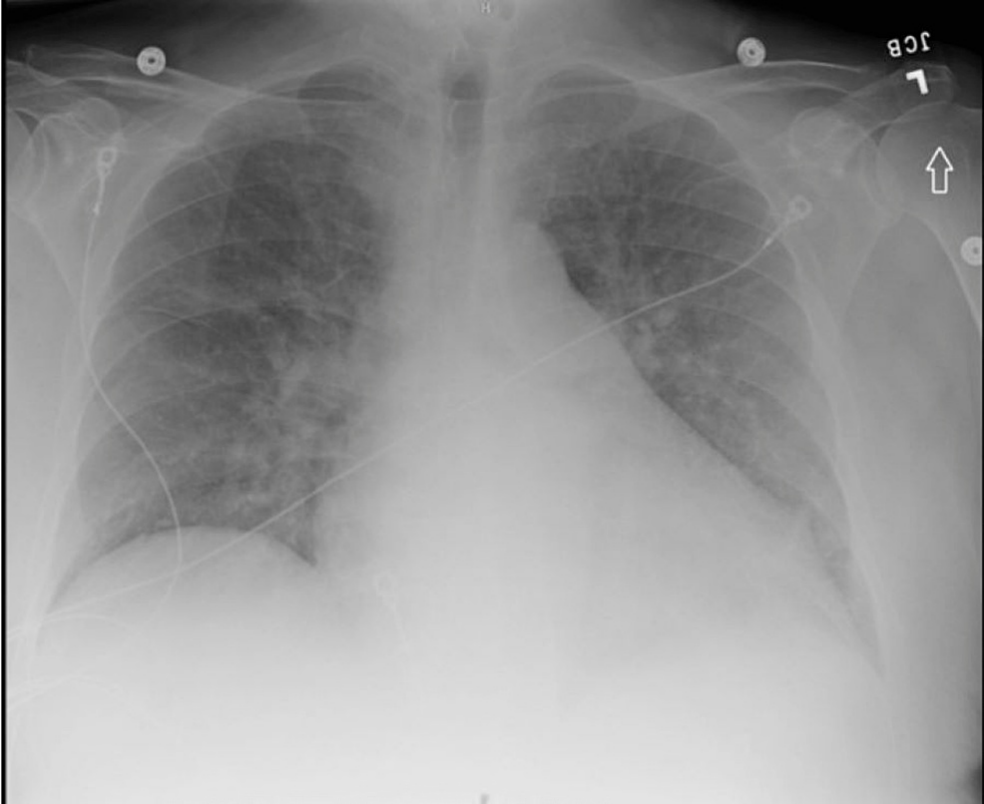
Chest radiograph showing cardiomegaly with pulmonary edema

**Figure 2:**
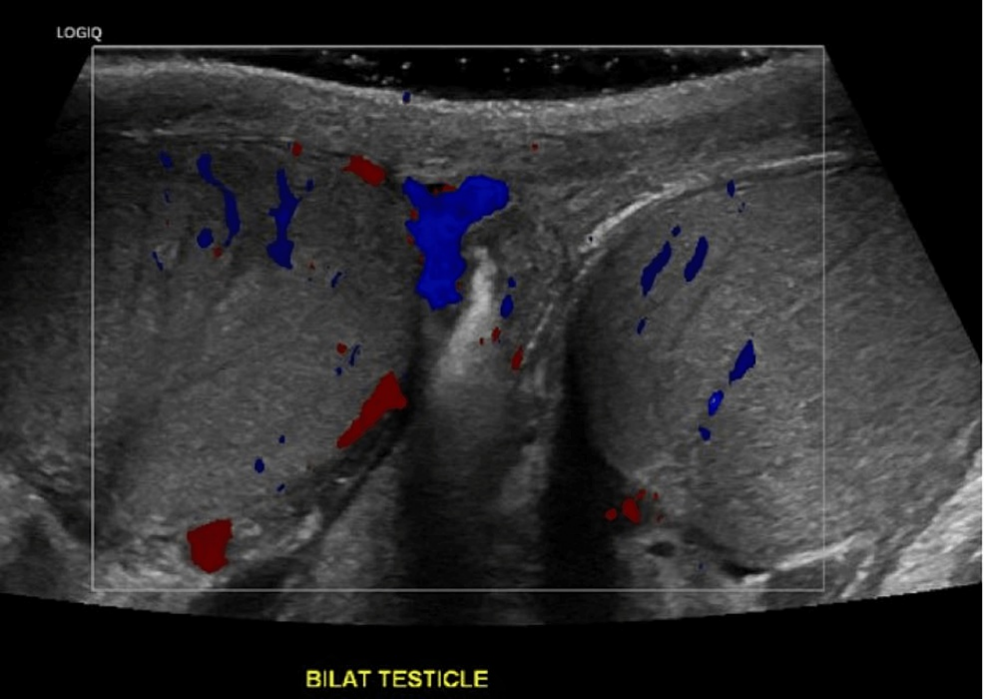
Testicular ultrasound showing homogeneous texture and normal Doppler flow

Laboratory findings are demonstrated in Table 1. Urinalysis was not sent due to anuria. Notably, he had mild uremia and tested positive for COVID-19. He also had anemia which was stable compared to baseline. Imaging included a chest radiograph (Figure 1) showing mild to moderate cardiomegaly with mild pulmonary edema. Testicular ultrasound was also obtained (Figure 2) and revealed non-specific, small bilateral hydroceles but was otherwise normal. CT scan of the abdomen and pelvis without contrast showed no specific etiology of his pain.

**Table 1:** Laboratory results BUN: Blood urea nitrogen

LAB TEST	VALUE	REFERENCE RANGE
WBC	5.4 K/uL	4.5-11 K/uL
Hemoglobin	10.5 g/dL	13.5-18 g/dL
Hematocrit	32.4 %	40.0-52.0%
Platelets	152 K/uL	140-440 K/uL
Glucose	105 mg/dL	70-99 mg/dL
Sodium	133 mEq/L	136-145 mEq/L
Potassium	4.8 mEq/L	3.6-5 mEq/L
Chloride	93 mEq/L	98-107 mEq/L
Carbon Dioxide	32 mEq/L	21-32 mEq/L
BUN	37 mg/dL	7-18 mg/dL
Creatinine	7.45 mg/dL	0.7-1.3 mg/dL
Coronavirus COVID-19 PCR	Detected	None Detected

The patient received doses of intravenous fentanyl and hydromorphone for pain control and hydralazine for blood pressure management. His systolic blood pressure was then documented to 200 mmHg, and he began to complain of pain radiating down his right leg. Nicardipine infusion was initiated, and a CT angiogram of the chest, abdomen, and pelvis was obtained due to concern for possible vascular pathology. This was negative for acute aortic dissection.

**Figure 3:**
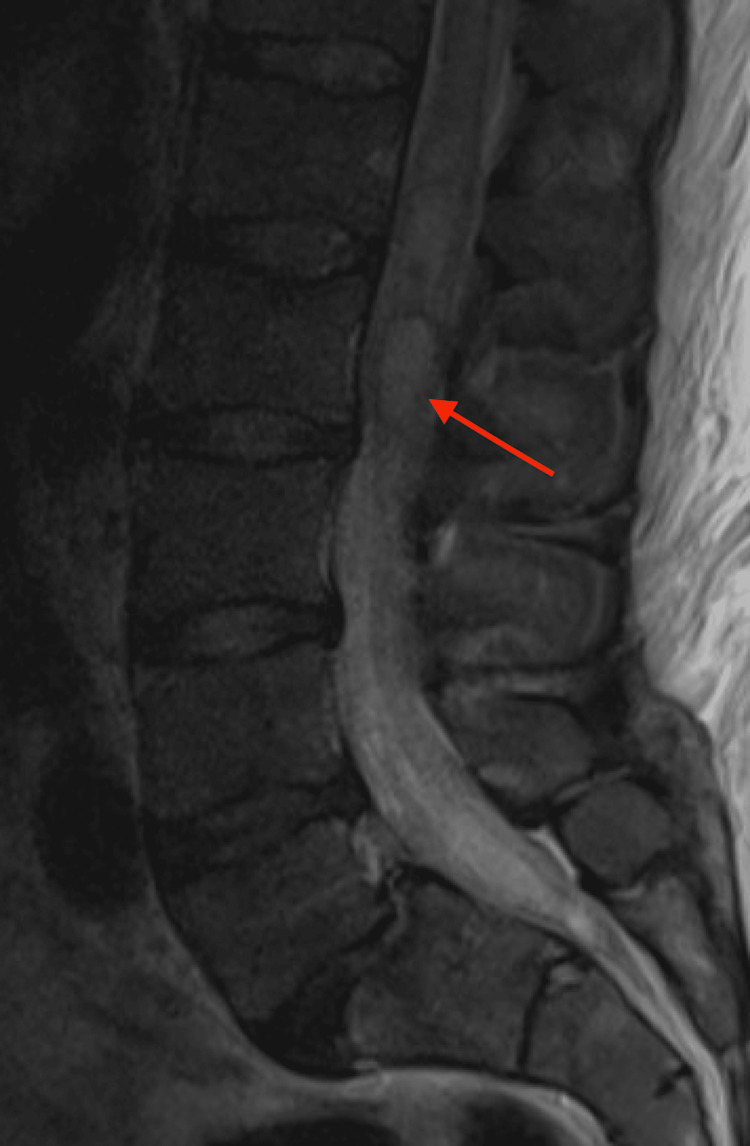
Sagittal lumbar spine MRI T2 image showing multilobulated, hyperintense mass associated at the level of L1-L3 measuring approximately 1.1 cm AP by 0.9 cm by 6.2 cm

The patient was admitted to the intensive care unit (ICU) for concern of possible hypertensive crisis as well as for ongoing monitoring and pain control. Upon admission to the ICU, he began to complain of right-sided leg numbness and weakness that progressed to left-sided leg numbness and weakness. Emergent lumbar spinal MRI was obtained (Figure 3) and illustrated an intramural mass at the level of L1-L3 with findings concerning possible hemorrhage.

Neurosurgery was consulted, and the patient was taken for diagnostic and therapeutic laminectomy, which revealed sSDH. His blood pressure was eventually controlled with oral medications, and he regained the majority of function in both of his legs with improvement in strength and sensation nearly to baseline. He did not develop any symptoms otherwise attributed to COVID-19. After 12 days of hospitalization, he was transferred to a rehabilitation facility for ongoing care.

## Discussion

This patient presented to the ED initially with the sole complaint of scrotal pain; however, his clinical course evolved to include radiating pain, numbness, and weakness in his legs, which we suspect was related to eventual hematoma expansion. His initial, isolated pain can be explained by spinal cord compression from the spontaneous sSDH at L1-L2 where nerve roots give origin to the ilioinguinal nerve and genitofemoral nerves supplying the scrotum [4,6].

Spinal hematomas in general are uncommon, but spontaneous sSDH appears to be quite rare [7-10]. One recent case series documented only 150 cases in the literature [7], and multiple reviews note that the majority of spontaneous bleeds are related to coagulopathy, especially anticoagulant use [7,8,11]. In nearly 90% of cases, motor deficits are the presenting exam abnormality, while sensory deficits occur in 64% [8]. Rettenmaier described hypertension as the most common comorbid medical condition - occurring in up to 16% of patients - and back pain as the most common presenting symptom [10]. Few cases of spontaneous sSDH occur without risk factors [17], and we believe that this patient's hypertension and ESRD were risk factors. It is not clear if his COVID-19 infection was an associated risk factor.

Interestingly, a few recent cases have demonstrated spontaneous spinal hemorrhage, including epidural and intramedullary hematomas with concomitant COVID-19 infection [12-15]. Only one case report discussing spontaneous sSDH with concurrent COVID-19 infection was found in our search [16]. That case described a 71-year-old female presenting with gait instability, thoracic pain, and left leg weakness associated with acute sSDH involving spinal levels C7-T3. The patient underwent laminectomy without complication; however, she did not regain baseline neurologic function.

Because of its rarity, diagnosing sSDH is challenging, but delays in diagnosis can lead to increased morbidity [9,11]. Furthermore, a comprehensive review by Vastani describes MRI as the gold standard for diagnosis of sSDH due to its ability to differentiate intradural hematoma as well as identify any vascular or neoplastic lesions [11]; yet MRI can be cumbersome or not available in many institutions. Additionally, Vastani notes that the most important signal of poor prognosis is the degree of neurologic deficit at presentation, highlighting the importance of early diagnosis. Unfortunately, ongoing neurologic deficits can occur in up to 28% of patients with sSDHs [9].

As mentioned, the majority of sSDHs are present in the thoracic region [8,10], most often causing thoracic back pain and sensory or motor deficits derived from the thoracic distribution. Nerve compression at the L1-L2 level, however, can present as isolated scrotal pain due to the somatic nerve supply to this area [4-6]. We believe this is the first case of spontaneous sSDH presenting as acute scrotal pain and a rare report of spontaneous sSDH with concomitant diagnosis of COVID-19 infection.

## Conclusions

While diagnosis of spontaneous sSDH in the ED may be extremely difficult, understanding various presentations and predisposing factors can lead to a more expedited diagnosis and treatment. We present a rare case of spontaneous sSDH presenting to the emergency room with acute scrotal pain. The presentation of acute scrotal pain in the ED includes a differential diagnosis with many high-risk etiologies. If the initial workup is negative and ongoing pain or other abnormalities are present, it is important to continue to widen the differential as acute spinal cord pathology can cause isolated scrotal pain as our case describes.
